# High HIV prevalence among decedents received by two high-volume mortuaries in Kisumu, western Kenya, 2019

**DOI:** 10.1371/journal.pone.0253516

**Published:** 2021-07-01

**Authors:** Dickens O. Onyango, Marianne A. B. van der Sande, Paul Musingila, Eunice Kinywa, Valarie Opollo, Boaz Oyaro, Emmanuel Nyakeriga, Anthony Waruru, Wanjiru Waruiru, Mary Mwangome, Teresia Macharia, Peter W. Young, Muthoni Junghae, Catherine Ngugi, Kevin M. De Cock, George W. Rutherford

**Affiliations:** 1 Kisumu County Department of Health, Kisumu, Kenya; 2 Ministry of Health, Nairobi, Kenya; 3 Department of Public Health, Institute of Tropical Medicine, Antwerp, Belgium; 4 Julius Global Health, Julius Centre for Health Sciences and Primary Care, University Medical Centre, Utrecht, Netherlands; 5 Division of Global HIV & TB (DGHT), US Centres for Disease Control and Prevention, Nairobi, Kenya; 6 Kenya Medical Research Institute (KEMRI), Kisumu, Kenya; 7 Global Programs for Research and Training, Nairobi, Kenya; 8 Ministry of Health, National AIDS and STI Control Program (NASCOP), Nairobi, Kenya; 9 Institute for Global Health Sciences, University of California, San-Francisco, California, United States of America; Thomas Jefferson University, UNITED STATES

## Abstract

**Background:**

Accurate data on HIV-related mortality are necessary to evaluate the impact of HIV interventions. In low- and middle-income countries (LMIC), mortality data obtained through civil registration are often of poor quality. Though not commonly conducted, mortuary surveillance is a potential complementary source of data on HIV-associated mortality.

**Methods:**

During April-July 2019, we assessed HIV prevalence, the attributable fraction among the exposed, and the population attributable fraction among decedents received by two high-volume mortuaries in Kisumu County, Kenya, where HIV prevalence in the adult population was estimated at 18% in 2019 with high ART coverage (76%). Stillbirths were excluded. The two mortuaries receive 70% of deaths notified to the Kisumu East civil death registry; this registry captures 45% of deaths notified in Kisumu County. We conducted hospital chart reviews to determine the HIV status of decedents. Decedents without documented HIV status, including those dead on arrival, were tested using HIV antibody tests or polymerase chain reaction (PCR) consistent with national HIV testing guidelines. Decedents aged less than 15 years were defined as children. We estimated annual county deaths by applying weights that incorporated the study period, coverage of deaths, and mortality rates observed in the study.

**Results:**

The two mortuaries received a total of 1,004 decedents during the study period, of which 95.1% (955/1004) were available for study; 89.1% (851/955) of available decedents were enrolled of whom 99.4% (846/851) had their HIV status available from medical records and post-mortem testing. The overall population-based, age- and sex-adjusted mortality rate was 12.4 per 1,000 population. The unadjusted HIV prevalence among decedents was 28.5% (95% confidence interval (CI): 25.5–31.6). The age- and sex-adjusted mortality rate in the HIV-infected population (40.7/1000 population) was four times higher than in the HIV-uninfected population (10.2/1000 population). Overall, the attributable fraction among the HIV-exposed was 0.71 (95% CI: 0.66–0.76) while the HIV population attributable fraction was 0.17 (95% CI: 0.14–0.20). In children the attributable fraction among the exposed and population attributable fraction were 0.92 (95% CI: 0.89–0.94) and 0.11 (95% CI: 0.08–0.15), respectively.

**Conclusions:**

Over one quarter (28.5%) of decedents received by high-volume mortuaries in western Kenya were HIV-positive; overall, HIV was considered the cause of death in 17% of the population (19% of adults and 11% of children). Despite substantial scale-up of HIV services, HIV disease remains a leading cause of death in western Kenya. Despite progress, increased efforts remain necessary to prevent and treat HIV infection and disease.

## Introduction

Although estimated deaths from human immunodeficiency virus (HIV) disease have been declining globally since 2006 [1], HIV remains a serious public health problem, especially in low-and medium-income countries (LMIC) of Africa. In 2018, an estimated 770,000 deaths were attributed to HIV infection, including 310,000 in eastern and southern Africa, and specifically, 25,000 in Kenya [2]. In 2016, United Nations member states made a political commitment to reduce mortality from HIV by 75% between 2010 to 2020 [3]. By 2018, no country was on track to achieving the United Nations HIV mortality goal [4].

Estimates from the Kenya National AIDS and Sexually Transmitted Infections (STI) Control Program (NASCOP) indicated that HIV prevalence among adults aged 15–49 years reduced from 7.1% in 2007 [5] to 5.6% in 2012 [6], and to 4.9% in 2019 [7]. While the decline in prevalence has been attributed to reduced HIV transmission due to high antiretroviral therapy (ART) coverage [6], the role of mortality in reducing HIV prevalence is unclear. Accurate data on HIV-associated mortality can help evaluate the impact of HIV interventions and progress in HIV prevention and control [8]. However, data on HIV-associated mortality are not generally available in LMIC, including Kenya, where the quality of civil registration and vital statistics is low, and underreporting of deaths is substantial [9–11]. Furthermore, causes of death, even when established, are not systematically recorded by many routine death-reporting systems [12–15]. HIV testing of decedents received by mortuaries offers a way of gaining further insight into HIV-associated mortality.

The HIV epidemic in Kenya is heterogeneous across the country’s 47 counties. HIV prevalence among people aged 15 years and above in Kisumu County (17.5%) is more than three times greater than the national prevalence [7]. In 2018, Kisumu County accounted for 6% of the estimated 28,200 HIV-associated deaths that are thought to have occurred in Kenya [16]. Kisumu was included among the nine of 47 counties that were responsible for 52% of Kenya’s HIV-associated deaths, although these counties accounted for only 28% of the country’s population [16].

Over the past three decades, studies in Cote d’Ivoire, the Democratic Republic of Congo, and Kenya have estimated HIV-associated mortality by testing adult decedents [17–20]. The feasibility of mortuary-based surveillance in Kenya was demonstrated in 2015 by a study of adolescent and adult decedents in the two largest mortuaries in Nairobi [21]. In that study, 19.5% of decedents were HIV-infected [19], and 65.7% of deaths among HIV-infected decedents were attributed to HIV [19]. Nairobi County has a substantially lower HIV prevalence (3.8%) than counties in western Kenya, where HIV prevalence is above 10% [7]. Systematically combining testing of decedents with unknown HIV status and abstraction of HIV status from medical records offers a way of assessing unexplored HIV-associated mortality. Given that the Nairobi study showed higher-than-expected mortality attributed to HIV, and poor coverage of diagnosis and treatment among HIV-infected persons dying, we undertook this investigation to estimate HIV-associated mortality in one of the highest HIV-burden regions of Kenya.

## Methods

This study was approved by KEMRI’s Science and Ethical Review Committee (#KEMRI/RES/7/3/1), Jaramogi Oginga Odinga Teaching and Referral Hospital (JOOTRH) Ethics Review Committee (#ERC.IB/VOL.1/615), and the University of California, San Francisco’s Committee on Human Research (#230355).

Informed consent was waived by the ethical review committees. This project was reviewed under CDC human research protection procedures. The CDC investigators did not interact with living human subjects or have access to identifiable data or samples for research purposes. Results of HIV testing were not linked back to living family or next of kin of decedents.

### Study setting

Kisumu County, located in the Nyanza region of western Kenya, had an estimated population of 1,155,574 in 2019 [22]. The city of Kisumu has 15 mortuaries, including those at JOOTRH and Kisumu County Referral Hospital (KCRH). These two mortuaries ordinarily receive more than 70% of decedents notified to the Kisumu East civil registry [23], which accounted for 45% of all deaths notified in Kisumu County in 2019. Over two thirds (70%) of decedents in the two mortuaries died in the host hospitals, while approximately 30% died in the community or other health facilities. In 2018, the estimated HIV prevalence in Kisumu County was 17.5% [7], translating to over 122,000 (9,439 children and 112,561 adults) people living with HIV (PLHIV) in the county, of whom 110,000 (90%) were diagnosed, and 105,000 (95% of the diagnosed) were on ART [24].

#### Study design

This was a cross-sectional study conducted in the two high-volume mortuaries attached to two referral hospitals in Kisumu County (JOOTRH and KCRH). Decedents admitted to the two mortuaries between April and July 2019 were consecutively enrolled. Data from the mortuaries were used to obtain medical records of decedents who died within the two hospitals. Chart reviews were done to establish if HIV infection was documented in medical records. Blood was collected by transthoracic cardiac puncture from decedents without documented HIV status in the medical records, including those dead on arrival, or whose latest HIV-negative result was older than three months. Death was considered to be due to HIV/AIDS if HIV or AIDS was listed as the underlying cause of death.

### Study population and population projections

All decedents with intact bodies, including children (0–14 years), received by the two mortuaries between April through July 2019 were eligible for enrollment into this study. We excluded decedents from whom blood could not be collected due to deterioration, burns, or embalming, stillbirth, and those who had been dead for ≥48 hours.

The age and sex distribution of the county population was obtained from the 2019 Housing and Population Census Report [22]. The 2019 age and sex-specific HIV prevalence for Nyanza region were projected using Spectrum version 5.52b3 (Avenir Health, Glastonbury, Connecticut). These rates were then scaled to the County HIV prevalence estimate for Kisumu County for 2019 from the National AIDS Control Council [25] and used to project the number of people living with and without HIV by age and sex.

### Variables

Demographic variables included age, sex, place of birth, date of birth, date of death, and place of death (died within JOOTRH or KRCH, died in the community or other facilities and transferred to JOOTRH/KRCH after death). Clinical and laboratory variables collected for hospital deaths from paper-based medical records included date of admission, clinical diagnosis, date of diagnosis, time of death, cause of death (COD) based on postmortem results if available, sample collection date, HIV test results, and viral load (VL) (copies/milliliter [mL] of plasma).

#### HIV and viral load testing

For each decedent, a 6 mL non-clotted blood sample was collected and transferred into sterile ethylene diamine tetra-acetic acid (EDTA) sample tubes and transported in a cool box to the Kenya Medical Research Institute (KEMRI) HIV research laboratory in Kisumu within 4 hours of collection. HIV testing was conducted in compliance with the 2015 Kenya HIV Testing Services guidelines [26,27]. Samples from children aged <18 months were tested using polymerase chain reaction (PCR). Samples from decedents aged 18 months and above were tested using Determine^™^ HIV-1/HIV-2^®^ (Abbott Diagnostic Division, Hoofddorp, Netherlands) as the screening assay, and First Response^®^ (Premier Medical Corp. Lt, Daman, India) as the confirmatory test. Samples that were reactive on Determine^®^ and First Response^®^ were considered positive. Samples that were reactive on Determine^®^ and non-reactive on First Response^®^ were considered discordant and retested by a different technician. After retesting, samples that remained discordant were tested using reverse transcriptase PCR (RT-PCR) to confirm the final HIV status. HIV-positive samples were further tested for viral load by RT-PCR using the Abbott^TM^ system (Abbott Molecular, Inc., Des Plaines, IL). The use of rapid test kits in this study is supported by previous studies showing that HIV antibodies remain detectable in serum for up to 58 days after death [28,29] and that performance of rapid HIV tests is comparable to that of standard enzyme immunoassay [28,30–32].

### Data analysis

Primary outcomes were the prevalence of HIV infection among decedents, attributable fraction among the HIV-exposed, and the population attributable fraction. We calculated the overall HIV prevalence among decedents stratified by age, sex, and place of death. HIV-associated mortality was defined as death in an HIV-infected person documented in the medical records or confirmed through HIV testing. We adjusted the number of deaths observed over the study period (87 days) to estimate the number of county deaths expected over 365 days by multiplying them by 365/87 and adjusting for coverage (the proportion of expected county mortalities observed in the study). Mortality rates were calculated per 1000 people by dividing the annualized deaths by the 2019 population for Kisumu County by HIV status, age, and sex. Pooled HIV positivity and mortality rates were then computed weighted by the projected population size by age, sex and HIV status.

The standardized mortality rate difference and standardized mortality rate ratio were calculated using Stata’s epitab package (Stata Corporation, College Station, Texas, USA). The standardized mortality rate difference was calculated by subtracting mortality rates in the HIV-uninfected population from mortality in the HIV-infected population standardized to the HIV-infected population by age and sex. To obtain SMRR, we divided mortality rates for HIV-infected decedents by mortality rates of HIV-uninfected decedents standardized to the age and sex distribution of the HIV-infected population. Attributable fraction among the exposed (HIV-infected decedents) and population attributable fraction were calculated by dividing standardized mortality rate difference by mortality rates of HIV-infected decedents and mortality rates in the population, respectively [33]. We tested for interactions between age and sex using logit models. We conducted Monte Carlo simulations to assess the sensitivity of obtained risk ratios and attributable fractions to various epidemiological assumptions. All statistical tests were done at 5% level of significance.

## Results

### Overview of decedents’ place of death and determination of HIV status

**Fig 1:**
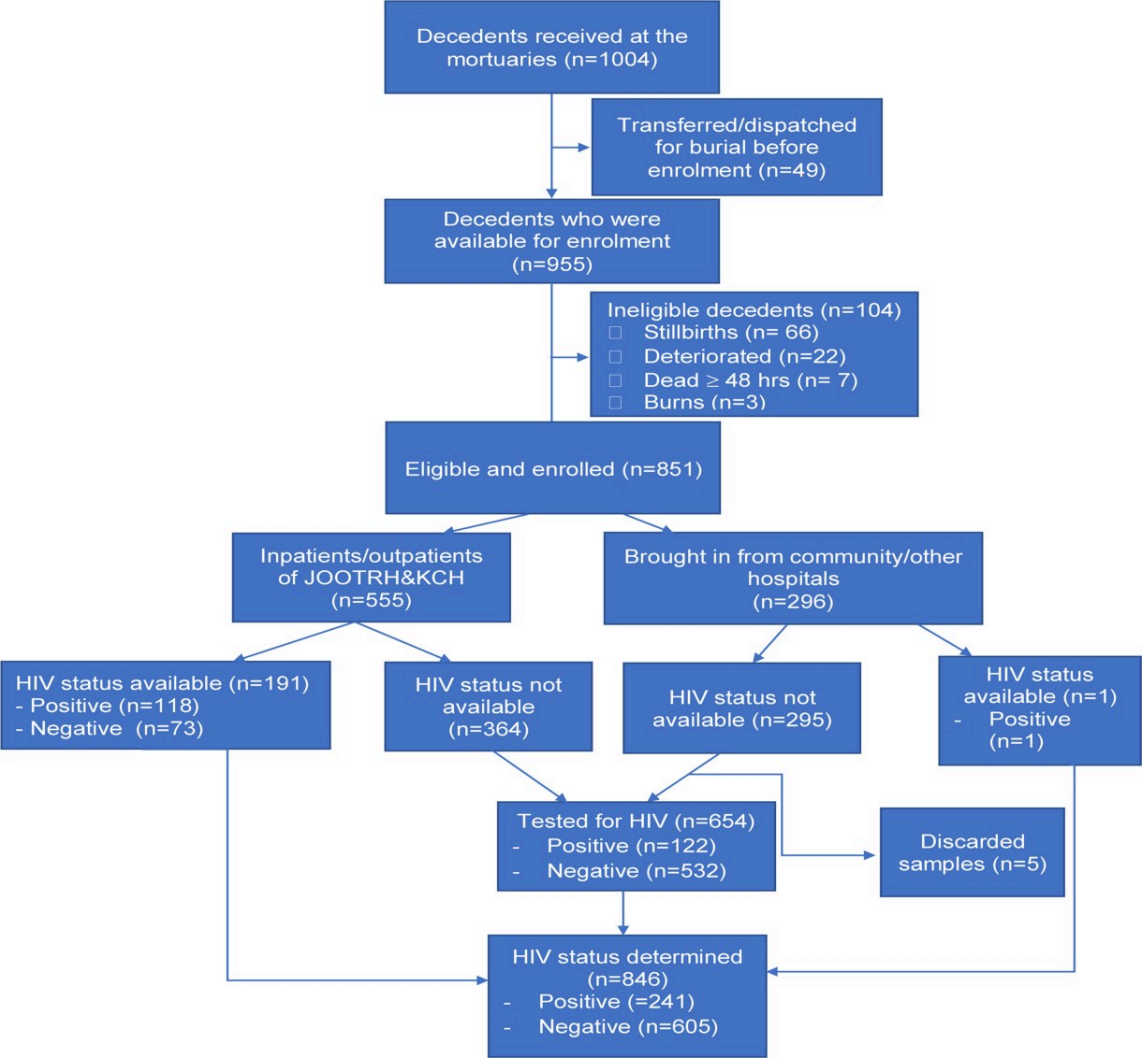
Flowchart depicting the source of decedents received by JOOTRH and KCH mortuaries, eligibility and source of their HIV status, Kisumu County, 2019.

Of 1,004 decedents received by the two mortuaries during the study period, 95.1% (955/1,004) were potentially available (not transferred out and not dispatched for burial before enrolment) for the study, 89.1% (851/955) of whom were enrolled (Fig 1). Of 104 ineligible decedents, 66 (63.5%) were stillbirths. Of the 851 decedents enrolled, 555 (65.2%) had died in JOOTRH or KCRH, and 296 (34.8%) were dead on arrival. Of the 555 decedents who had died within JOOTRH or KCRH, 34.4% (191/555) had HIV status documented in the chart; 61.8% (118/191) were known to be HIV-positive. Of the 296 decedents who were dead on arrival, 1% (1/296) was known to be HIV-positive. Blood samples were drawn from the remaining 659 decedents with unknown HIV status (364 who had died in JOOTRH or KCRH and 295 who were dead on arrival); <1% (5/659) had untestable blood specimens. Of the 654 samples tested, 18. 7% (122/654) were HIV-positive. HIV positivity was 14.8% (54/364) among tested decedents who died in hospital and 23.4% (68/290) among tested decedents who were were dead on arrival. Overall, 846 decedents had their HIV status determined, 192 through documentation, and 654 through post mortem HIV testing. Thus, 28.5% (241/846) overall were determined to be HIV infected (119 by medical records review and 122 by testing). The the age and sex adjusted pooled HIV prevalence was 26.3% (95% CI: 23.4–29.4) (Table 1). The adjusted HIV prevalence was higher among females (29.0%; 95% CI: 24.8–33.5) than males (24.1%; CI 20.3–28.3) (p-value = 0.05).

**Table 1:** Unadjusted and adjusted HIV prevalence among Kisumu decedents by age and sex (N = 846).

Sex	Age group (Years)	Number of deaths	Unadjusted Positivity % (95% CI)	Age and Sex Adjusted Positivity* % (95% CI)
Male	0–14	76	13.16 (7.19–22.85)	-
	15–24	33	21.21 (10.33–36.62)	-
	25–44	145	37.93 (30.37–46.12)	-
	45+	182	21.98 (16.53–28.61)	-
	Total	436	25.69 (21.80–30.00)	24.06 (20.27–28.32)
Female	0–14	84	11.90 (6.50–20.81)	-
	15–24	25	40.00 (22.74–60.17)	-
	25–44	101	65.35 (55.52–74.02)	-
	45+	200	21. 50 (16.33–27.76)	-
	Total	410	31.46 (27.14–36.13)	28.98 (24.81–33.54)
	Grand total	846	28.49 (25.54–31.63)	26.28 (23.42–29.36)

* Positivity adjusted by expected age-sex distribution among decedents according to population projection.

#### Demographic characteristics of enrolled decedents

Children accounted for 18.9% (161/851) of enrolled decedents. About half, 49.7% (80/161) of decedent children were under 1 year old, 28.0% (45/161) were aged 1 to 4 years, while the age groups 5 to 9 and 10 to 14 years each accounted for 11.2% (18/161). Most decedent children, 82.0% (132/161), were inpatients in the two hospitals before death, 16.2% (26/161) were dead on arrival, and 1.2% (2/161) died in the outpatient departments. One decedent 0.6% (1/161) was brought in as a police case.

Adolescents and adults accounted for 81.1% (690/851) of enrolled decedents; 9.9% (58/690) of adolescent and adult decedents were aged 15 to 24 years, 19.0% (131/690) were aged 25 to 34 years, 17.0% (117/690) were 35 to 44 years old, and 55.7% (384/690) were aged 45 years and above. Males accounted for 52.5% (362/690) of decedent adolescents and adults. Most adolescent and adult decedents, 54.8% (378/690), were inpatients in the two hospitals, 33.0% (228/690), were dead on arrival, 6.2% (43/690) died within outpatient departments, and 5.9% (41/690) were police cases.

### HIV prevalence, treatment, and viral suppression

Among the 119 decedents with documented HIV-infection, 58.8% (70/119) had medical records indicating they were on ART during the hospitalization preceding death, 16.0% (19/119) were not, and 25.2% (30/119) had no documented ART status (S1 Fig). Among the 122 decedents who were HIV-positive through testing, viral load (VL) was available for 96.7% (118/122); one sample returned invalid results while four samples were insufficient. The median VL for the 117 decedents was 839 copies per milliliter (copies/mL) of plasma (interquartile range (IQR): 40–37,526); 49.6% (58/117) of decedents with VL results had copies above 1,000 copies/mL. The median viral load among decedent children was 322,911 copies/mL (IQR: 147, 371–578, 504 copies/mL); 100% (11/11) of children with VL had copies above 1,000 copies/mL.

### HIV-related mortality rates and attributable fraction

According to the 2019 Kenya Housing and Population Census, the population of Kisumu County in 2019 was 1,155,574 [22]. Using Spectrum (Avenir Health, Glastonbury, Connecticut), we estimated 83,085 people were living with HIV. We projected that 14,352 deaths occurred in the county in 2019, giving an overall mortality rate of 12.4 per 1,000 population (Table 2). The overall mortality rate was higher in males (13.1 per 1,000 population) than females (11.8 per 1,000 population) (p-value<0.001). The mortality rate was four times higher in the HIV-infected (40.7 per 1000) than the HIV-uninfected population (10.2 per 1,000) (p-value<0.001). The mortality rate in the HIV-infected population was 14.2% higher in males (44.3 per 1,000 population) than females (38.0 per 1,000 population) (p-value<0.001). Male and female children (0–14 years) and adults aged 45 or older had higher death rates than persons aged 15–34 and 35–44. The overall unadjusted mortality rate ratio (HIV-infected versus HIV–uninfected) was 4.0; the mortality rate ratio was highest among male children (12.8), followed by female children (11.4).

**Table 2:** Mortality rates by age, sex and HIV status, Kisumu County, 2019.

		HIV-uninfected			HIV-infected				Overall		
Sex	Age group (Years)	Deaths	Pop.	Mortality Rate (per 1,000 pop)	Deaths	Pop.	Mortality Rate (per 1,000 pop)	Mortality Rate Ratio (HIV-infected/-uninfected)	Deaths	Pop.	Mortality Rate (per 1,000 pop)
Male	0–14	2,497	223,104	11.2	378	2,645	142.9	12.8	2,875	225,749	12.7
	15–24	493	113,399	4.4	133	3,869	34.4	7.9	626	117,268	5.3
	25–44	788	130,210	6.1	481	17,057	28.2	4.7	1,269	147,267	8.6
	45+	2,024	58,979	35.3	570	11,666	48.9	1.4	2,594	70,646	36.7
	**Total**	**5,802**	**525,691**	**11.0**	**1,562**	**35,237**	**44.3**	**4.0**	**7,364**	**560,928**	**13.1**
Female	0–14	2,237	224,836	10.0	302	2,672	113.0	11.4	2,539	227,508	11.2
	15–24	345	123,028	2.8	230	7,648	30.1	10.7	575	130,676	4.4
	25–44	359	129,604	2.8	677	24,139	28.1	10.1	1,036	153,743	6.7
	45+	2,228	69,278	32.2	610	13,389	45.6	1.4	2,838	82,667	34.3
	**Total**	**5,169**	**546,746**	**9.5**	**1,819**	**47,848**	**38.0**	**4.0**	**6,988**	**594,594**	**11.8**
**Grand Total**		**10,971**	**1,072,437**	**10.2**	**3,381**	**83,085**	**40.7**	**4.0**	**14,352**	**1,155,522**	**12.4**

Adjusted for age and sex, the mortality rate among PLHIV was thrice higher than that among HIV-uninfected people (standardized mortality rate ratio = 3.1; 95% CI: 2.60–3.64) (Table 3). The standardized mortality rate ratio was marginally higher in females (3.32; 95% CI: 2.63–4.20) than males (2.84; 2.23–3.61). Adjusted for age and sex, there were 28 additional deaths annually for every 1,000 people among PLHIV (standardized mortality rate difference = 0.028; 95% CI: 0.022–0.033), 29 for men and 27 for women. Overall, the attributable fraction among the exposed was 0.71 (95% CI: 0.66–0.76); the attributable fraction among the exposed was 1.4 times higher in children <15 years (0.92; 95% CI 0.89–0.94) than in adolescents and adults (0.64; 95% CI: 0.56–0.69). The overall population attributable fraction was 0.17 (95% CI = 0.14–0.20); the population attributable fraction was 1.7 times higher in adolescents and adults (0.19; 95% CI: 0.14–0.24) than in children (0.11; 95% CI: 0.08–0.15).

**Table 3:** Standardized mortality ratios, risk difference and attributable fractions, Kisumu County, 2019.

Indicator	Sex		Total
	Male	Female	
**Children (0–14)**			
SMRR	12.53 (8.24–19.06)	11.53 (7.22–18.39)	12.06 (8.83–16.47)
SMRD	0.130 (0.075–0.184)	0.104 (0.054–0.154)	0.117 (0.080–0.154)
AFe	0.92 (0.88–0.95)	0.91 (0.86–0.95)	0.92 (0.89–0.94)
AFp	0.12 (0.07–0.17)	0.11 (0.06–0.16)	0.11 (0.08–0.15)
**Adolescents & Adults (15+)**			
SMRR	2.28 (1.74–3.00)	2.90 (2.25–3.74)	2.59 (2.15–3.12)
SMRD	0.020 (0.012–0.029)	0.022 (0.015–0.029)	0.021 (0.016–0.027)
AFe	0.58 (0.45–0.68)	0.67 (0.57–0.74)	0.64 (0.56–0.69)
AFp	0.15 (0.09–0.21)	0.22 (0.16–0.29)	0.19 (0.14–0.24)
**Overall**			
SMRR	2.84 (2.23–3.61)	3.32 (2.63–4.20)	3.08 (2.60–3.64)
SMRD	0.029 (0.020–0.038)	0.027 (0.020–0.034)	0.028 (0.022–0.033)
AFe	0.69 (0.61–0.76)	0.73 (0.66–0.78)	0.71 (0.66–0.76)
AFp	0.15 (0.10–0.19)	0.19 (0.14–0.23)	0.17 (0.14–0.20)

SMRR, standardized mortality rate ratio; SMRD, standardized mortality rate difference; AFe, attributable fraction in the exposed; AFp, population attributable fraction.

## Discussion

Data on mortality due to HIV can help evaluate the impact of ART programs and guide efforts to strengthen HIV care. Despite the high ART coverage among PLHIV documented through population-based surveys [7], we determined that nearly one-third of decedents in Kisumu were HIV-infected. This analysis demonstrates that 17% of mortality in the western Kenya population could be prevented through early identification of PLHIV, prompt ART initiation, and strengthening adherence to ART. It is a cause for concern that only about one third of in-hospital decedents had their HIV status documented in their medical records; that about 15% of hospital decedents with undocumented HIV status were actually HIV-infected; and that about one quarter of those who were dead on arrival were HIV-positive. The high proportion of decedents who were virally non-suppressed together with a high median viral load (839 copies/mL) suggest that many may not have been on ART or had failed treatment. Clearly, increased effort is required to diagnose all persons with HIV and assure rapid initiation of ART, adherence to treatment, and appropriate laboratory monitoring [34]. Work is also required to understand apparent differences between the uptake of diagnostic testing and ART among decedents documented by this study, and results from HIV program evaluations which suggest 90% of admitted patients are tested for HIV.

The adjusted HIV positivity among decedents in this study was higher than observed in Nairobi [35] but lower than studies conducted during the pre-ART era in other high HIV prevalence settings such as the Democratic Republic of Congo [18] and Cote d’Ivoire [36]. The higher positivity in decedents in Kisumu compared to Nairobi could be explained by differences in overall population HIV prevalence. According to the Kenya Population Population-based HIV Impact Assessment (KENPHIA) 2018, HIV prevalence in the adult population was five times higher in Kisumu County (17.5%) than in Nairobi County (3.8%) [7]. HIV prevalence among decedents whose HIV status was obtained from medical records was many times higher than the population prevalence, likely because patients with serious HIV disease are concentrated in hospitals [36]; by contrast, the prevalence among those who were tested in the study was similar to the population prevalence. This is supported by evidence from other sub-Saharan countries where HIV has been reported to be a leading cause of hospitalization [37,38] and inpatient mortality [39,40].

In this study, the first of its kind to include children from a high HIV prevalence setting, children living with HIV were over ten times more likely to die than their HIV-uninfected counterparts. The attributable fraction among the exposed, above 90%, indicates that HIV was the cause of death in most of these children. Previous studies have documented a larger gap in HIV diagnosis, ART initiation, and viral suppression in children than in adults [41–44]. Late HIV diagnosis [45] and high loss to follow-up after ART initiation [46] are important contributors to mortality in HIV-infected children. All the decedent children with viral load results were unsuppressed, indicating that they were possibly not on ART, had just recently initiated ART, were not adhering to ART, were on a sub-optimal regimen, or were experiencing treatment failure. We could not establish whether children under one year were being followed up for being HIV exposed. Over the last decade Kenya has accelerated efforts to control the pediatric HIV epidemic through intensified identification of HIV-infected children and ART initiation. In an evaluation of the United Nations Joint Program on HIV/AIDS (UNAIDS) 90-90-90 targets using KENPHIA data, 78.9% of HIV-infected children had been identified, 93.2% of whom were on ART, and 67.1% of whom were virally suppressed [7]. Just as for adults, the optimistic assessment of program quality from population-based surveys fails to capture mortality data and related insights into diagnosis and treatment. Greater effort is required to diagnose, treat, and ensure viral suppression among HIV-infected children.

The population attributable fraction for adolescents and adults observed in our study was similar to that reported by the Nairobi mortuary study (17% vs. 16%) [35]. The similarity in population attributable fraction despite large differences in HIV prevalence could be due to enhanced implementation of ART programs over the intervening years, especially in high prevalence counties. Global Burden of Disease data reported larger declines in HIV-specific mortality in counties with the highest burden of HIV [47]. Nonetheless, despite high ART coverage, estimated at 76% for all persons with HIV in the 2018 population-based survey [7], HIV remains a leading cause of mortality in this high HIV burden setting, with more than two-thirds of deaths in PLHIV attributed to HIV infection. Other studies have also reported higher mortality in HIV-infected people even in the era of ART [35], probably resulting from late ART initiation [48–52], pretreatment drug resistance, treatment failure arising from poor adherence, and treatment interruptions [53]. Early identification of PLHIV and ART initiation could substantially dent the impact of HIV on mortality in high prevalence settings [54,55].

Unlike the Nairobi study, which tested all decedents, our study explored the abstraction of HIV status from medical records, a more convenient approach to HIV surveillance among decedents. However, we noticed that documentation of HIV and ART status in medical records was poor. Although we successfully obtained HIV infection information for half of all HIV-infected decedents from medical records, critical information was missing. This may have been caused by the segmental nature of handling medical records in this setting. Often, HIV programs maintain electronic health records (EHR) within comprehensive care centers that are not readily available to other service delivery points such as inpatient departments. Expansion of EHRs to other service delivery points could increase the quality of HIV information available in the medical records and contribute to better patient outcomes.

Our study had several limitations. It was conducted exclusively in public-sector mortuaries. Not all deaths in the community are captured by mortuaries of any kind. Nonetheless, we believe our data are representative. Our two study hospitals receive 70% of decedents in Kisumu, with the other 30% shared by private and other public facilities. We used data from three months to estimate annual mortality. This approach may have introduced bias from seasonal differences in mortality. We utilized HIV status from medical records for hospital deaths when available as advised by the ethical review committee, but this precluded viral load testing for HIV-positive decedents thus identified. Abstracting ART status from medical records could have led to misclassification of decedents and testing of blood for ART metabolites was not possible. [56]. Obtaining HIV status from the medical records could have biased the estimates calculated in the study (HIV prevalence and attributable fractions). However, we minimized this by testing decedents whose documented HIV-negative status was older than three months.

### Conclusions

Our study documented a higher than expected prevalence of HIV infection among decedents received by two high-volume mortuaries in western Kenya. The majority of HIV-infected decedents with viral load measurements were not virally suppressed. The fraction of mortality in the population attributable to HIV infection was high, 17% (17%). Preventing HIV-attributable mortality could substantially reduce overall mortality in this high-HIV-prevalence population.

## Supporting information

S1 Fig ART status and viral suppression among HIV-infected decedents enrolled in the HIV mortality surveillance study, Kisumu County, 2019. * viral load were not abstracted from the inpatient/outpatient hospital records, they are usually recorded in comprehensive care center records that were not reviewed as part of this protocol.
(TIF)

S1 Data (DTA)
