# Supplementary figures and images for: High HIV prevalence among decedents received by two high-volume mortuaries in Kisumu, western Kenya, 2019
Source: PLoS One. 2021 Jul 1;16(7):e0253516. doi: 10.1371/journal.pone.0253516 (PMC8248726; doi:10.1371/journal.pone.0253516)

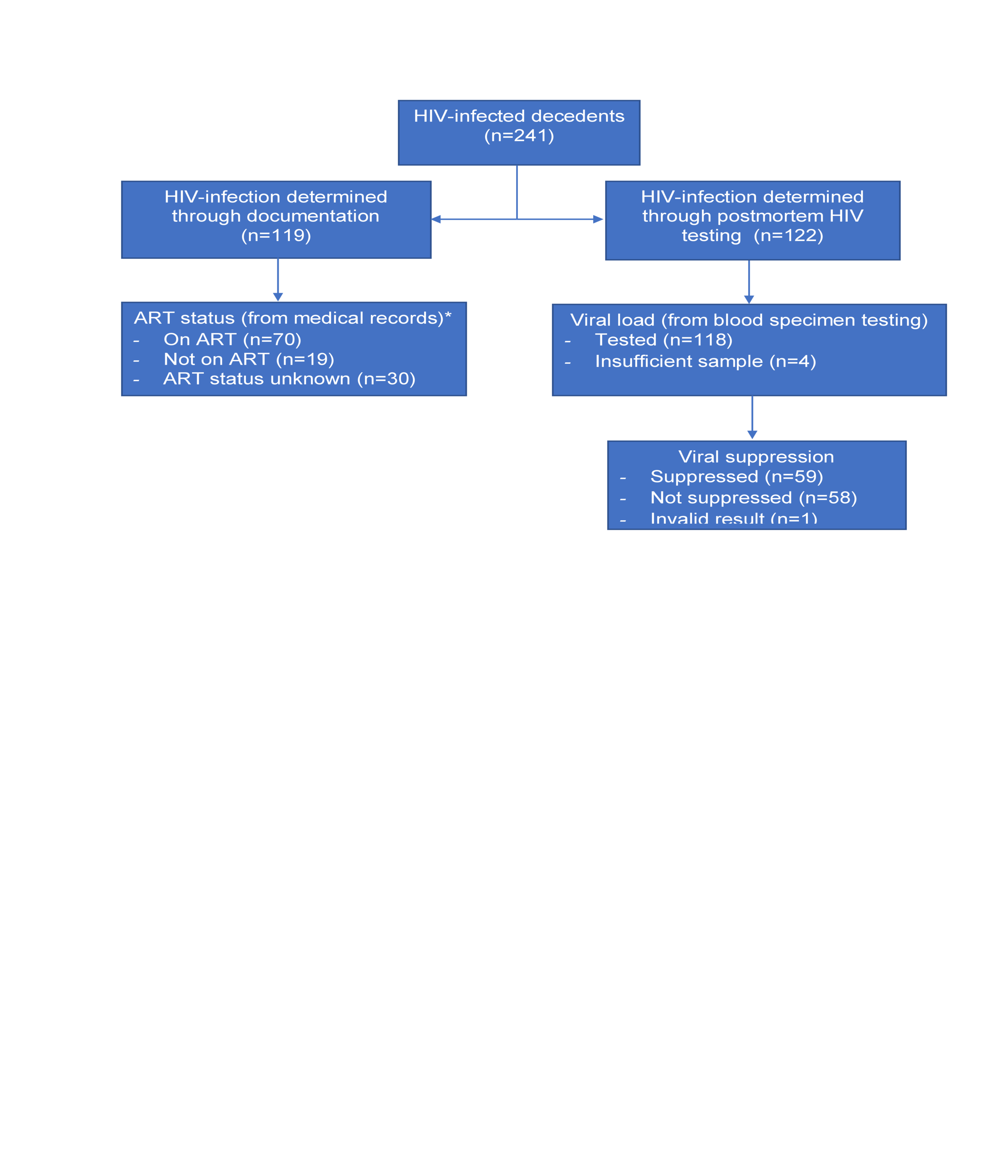

Supplement: S1 Fig — * viral load were not abstracted from the inpatient/outpatient hospital records, they are usually recorded in comprehensive care center records that were not reviewed as part of this protocol. (TIF) [file pone.0253516.s001.tif]
